# The influence of subjective social class on employment confidence: the chain mediating effect of perceived social support and self-efficacy

**DOI:** 10.1186/s40359-025-02861-3

**Published:** 2025-09-10

**Authors:** Youxia Zuo, Yongzheng Huang, Liping Mou, Bing Chen, Yujie Chen

**Affiliations:** 1 School of Literature, Southwest University, 400715 Chongqing, China; 2 Center for Studies of Psychological Application, South China Normal University, 510631 Guangzhou, China; 3 Key Laboratory of Brain, Cognition and Education Sciences, Ministry of Education, South China Normal University, 510631 Guangzhou, China; 4 School of Psychology, South China Normal University, 510631 Guangzhou, China; 5 Party Committee student work Department, Southwest University, 400715 Chongqing, China; 6 Faculty of Psychology, Southwest University, 400715 Chongqing, China

**Keywords:** Undergraduates, Employment confidence, Subjective social class, Perceived social support, Self-efficacy

## Abstract

With the persistence of difficult employment, a large number of college students feel anxious and nervous about job hunting. College students with different family economic status have various feelings and performances when faced with employment, possibly due to subjective social class differences. The present study investigated the employment confidence of 611 undergraduates in Chongqing, aimed to ascertain the overall employment confidence of Chinese college students, and tried to analyze how subjective social class works on the employment confidence of college students and its influencing mechanism. The results revealed that the overall employment confidence of college students was positive. There is no significant difference in employment confidence between males and females. The employment confidence of public-funded normal students is higher than that of other college students, and the employment confidence of freshmen is lower than that of juniors and sophomores. What’s more, the subjective social class could directly affect employment confidence and also could affect employment confidence by the mediating effect of perceived social support and self-efficacy. Further, subjective social class works on employment confidence through the chained mediating effect of perceived social support and self-efficacy. It suggested that it is worth paying attention to enhancing the perceived social support and self-efficacy of undergraduates to foster their employment confidence because of a relatively stable subjective social class.

**Supplementary Information:**

The online version contains supplementary material available at 10.1186/s40359-025-02861-3.

## Introduction

In recent years, the number of college graduates in China has reached record highs year after year, and the label of “the most difficult employment season” has been hard to shake off. Against the backdrop of a challenging global economic situation, phenomena such as “slow employment” and “lazy employment” have gradually emerged. How to boost the employment confidence of college students and improve their employment situation remains a continuous concern for education and society. Relevant studies have pointed out that one of the key reasons for employment difficulties is the lack of employment confidence [1–3]. Employment confidence represents individuals’ positive feelings about themselves in the process of job-hunting, which also means individuals’ belief and expectation that they could get an ideal job through their own efforts after considering various possible factors [4]. Building and maintaining employment confidence is of great significance for undergraduates to cope with employment pressure and maintain mental health. Whereas, previous researches have paid more attention to employment issue at the social macro level, and paid insufficient attention to the micro employment psychology, especially from the perspective of employment confidence. Even in the few studies that involve employment confidence, they were inclined to reveal the general situation of employment confidence or superficially to explore the effects of some factors on employment psychology, such as employability and family support [5, 6]. Current studies have failed to reveal the causes and mechanisms of college students’ inadequate confidence in employment after comprehensive consideration of social and individual factors. Being in the state of a certain social class would profoundly affect one’s employment psychology and behavior. The present study tried to explore the impact of subjective social class differences on college students’ employment confidence and its influencing mechanism, intending to provide suggestions for enhancing college students’ employment confidence, promoting their mental health, and facilitating their smooth employment.

### The relationship between subjective social class and employment confidence

Social class reflects individuals’ relative rank within the social hierarchy, which consists of both objective social class and subjective social class [7]. The former implies individuals’ possession of material and social resources and generally has been measured with objective indicators, such as income, education, and occupation. While subjective social class emphasizes individuals’ genuine perception of possession of social resources and rank relative to others in society [8]. Previous researches indicated that objective social class wasn’t consistent with subjective social class; as for psychological outcome variables, subjective social class seemed more predictive than objective social class in various domains, such as self-rated health and subjective well-being [9, 10] Therefore, the present study employed subjective social class to explore the impact of undergraduates’ subjective perception of social resources and status on employment confidence. The social cognitive theory of social class proposes that subjective social class leads to a coherent set of social cognitive tendencies and guides patterns of thought, feeling, and action [11]. Thus, we inferred that in the employment context, subjective social class also affects an individual’s employment confidence.

According to the social cognitive theory of social class, individuals with lower subjective social class are more likely to have their pursuit of goals and interests constrained by their reduced economic resources and social rank, compared to those with high subjective social class. These individuals exhibit a diminished sense of control and heightened threat sensitivity, demonstrating more pronounced stress responses and behavioral manifestations when confronted with ambiguously threatening social scenarios [11, 12]. The same is true in the employment environment. Due to the visible differences in family socioeconomic status, college students in various classes would display inequitable ability to utilize resources and endure pressure when they apply for jobs. Specifically, compared with the higher subjective social class, college students with lower subjective social class are less abundant in resources and are vulnerable to competition, who could perceive more deprivation and be in low self-worth; reversely, college students with a high subjective social class tend to experience psychological advantages stemming from favorable economic and educational conditions, which results in more positive self-perception, heightened job-seeking optimism, and enhanced resilience to setbacks [13, 14]. Thus, the present study assumed that subjective social class could positively predict employment confidence among undergraduates (hypothesis 1).

### The mediation of perceived social support

Perceived social support was considered as a potential mediator linking subjective social class to employment confidence, because subjective social class is intrinsically tied to resource access, and may shape individuals’ perceived social support through resource-driven mechanisms [11, 15]. The higher subjective social class, owing to their resource advantages, could access both more abundant instrumental support (e.g., economic and material resources) and greater psychosocial support, including respect, emotional care, and social recognition [16–18]. The main-effect model of social support posits that social support has a beneficial effect irrespective of whether persons are under stress. The buffering model of social support emphasizes that social support protects persons from the potentially negative influence of stressful events and enhances individual mental health [19]. Therefore, irrespective of whether they perceive employment as a source of pressure, college students from diverse social classes may develop distinct employment mindsets owing to variations in the quality of their social support. Perceived social support more accurately reflects the quality and effectiveness of social support, encompassing their expectations, evaluations, and beliefs regarding the potential social support they may receive [20–22]. Perceived social support frequently functions as a mediator in the relationship between subjective social class and psychological outcome variables because it influences an individual’s evaluative processes; that is, the subjective social class may affect individual behavior, cognition, and emotion through the function of perceived social support [23, 24]. Previous works revealed that the higher the subjective social class individuals occupied, the higher the level of perceived social support they could respond to [25, 26]. When confronted with challenges, individuals with a higher subjective social class are more likely to recognize the support from their social networks, experience a greater sense of being supported, and exhibit higher levels of self-confidence [27]. Given this, the study inferred that there was a mediating effect of perceived social support between subjective social class and employment confidence (hypothesis 2).

### The mediation of self-efficacy

Social class significantly influences an individual’s self-concept and cognitive tendencies. The lower social class tends to exhibit contextualist social cognitive tendencies, experience a diminished sense of control, and place less emphasis on personal abilities and values. Whereas individuals with a higher subjective social class are more likely to demonstrate solipsistic tendencies, an elevated sense of control, and a self-concept characterized by increased personal agency. The extant literature consistently corroborates the influence of social class on self-efficacy [28, 29]. Self-efficacy refers to an individual’s belief in their capability to execute a behavior successfully despite potential obstacles, which exerts a profound and sustained influence on an individual’s physical and mental development as well as behavioral outcomes [30–32]. Individuals naturally have self-efficacy, which can predict their general confidence in coping with pressures and challenges, and the exact embodiment of high self-efficacy is confidence [33, 34]. A study examining the connection between self-efficacy, academic performance, and employment confidence has affirmed that self-efficacy could effectively predicate employment confidence [35]. Simultaneously, another study among Chinese students found that the high self-efficacy undergraduates possessed satisfactory social adaptability and adequate employment confidence compared with those who had low self-efficacy [36]. Given this, we hypothesized that self-efficacy might play a mediating role in the relationship between subjective social class and employment confidence (hypothesis 3).

### The chain mediation of perceived social support and self-efficacy

Social Cognitive Theory posits that external expectations, along with the associated support and guidance, significantly influence an individual’s self-efficacy [37]. Adolescents’ self-efficacy could be influenced by their family’s socioeconomic status, and this effect worked primarily via their perceived family support. As a part of social support, family support particularly played a significant role in promoting students’ self-efficacy [17, 38]. The Family Stress Model indicates that a low socioeconomic status within a family has detrimental effects on both family functioning and individual adaptation. Consequently, children from economically disadvantaged families are at a higher risk of experiencing developmental challenges [39]. For college students with a lower subjective social class, their parents often struggle to provide adequate attention and support due to financial pressure within the family and limitations on time. Consequently, these students usually experience diminished feelings of family support, which in turn decreases their sense of self-efficacy. In contrast, college students from higher subjective social classes tend to perceive greater access to social resources and feel more family support, thereby enhancing their sense of self-efficacy [40]. Various researchers affirmed that perceived social support could make individuals feel that their requisite resources were more likely to be met and then improve an individual’s actual coping efficacy and self-efficacy [41, 42]. Thus, we put forward a hypothesis that subjective social class works on employment confidence through the chain mediating effect of perceived social support and self-efficacy (hypothesis 4).

**Fig. 1:**
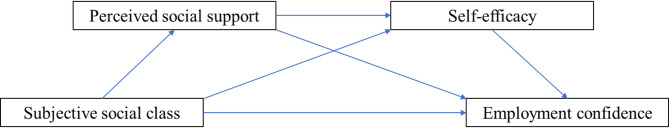
Hypothetical model

Taken together, the current research aimed to explore the specific circumstance of employment confidence among college students and examine the following chain mediating model based on the above-mentioned four hypotheses (Fig. 1). We hoped to reveal the influencing mechanism of subjective social class on undergraduates’ employment confidence, attempting to obtain enlightenments on the cultivation of employment confidence.

## Methods

### Participants

A total of 611 college students were responsive to the research by online survey. Among them, 11 participants with obvious perfunctory feedback were excluded, and 600 participants (404 women and 196 men; *M*_age_ = 20.067, *SD* = 1.06) in total provided effective and unabridged feedback for the survey. Furtherly, the whole effective subjects consisted of freshmen (*n* = 212), sophomores (*n* = 235), and juniors (*n* = 153). Considering that there were some psychological interference factors for senior students to participate in recruitment, no senior students were recruited. This study has been approved by the Ethics Committee of Southwest University. All participants were given the informed consent statement before the formal investigation and received remuneration for participating in this study.

### Measures

Subjective social class was measured by the MacArthur Scale of socioeconomic status [43]. The scale uses a 10-step ladder to represent different social classes, with the top “10” representing the highest social class, enjoying the highest income, the best education, the most decent job, and the best life; the “1” at the bottom represents the lowest social class, the worst life, the lowest income, and the worst job. The participants were asked to evaluate the current class of their family’s socioeconomic status on this ladder (10-point scale), and the corresponding number was used as the subjective social class score. The higher the score indicated a higher subjective social class.

Self-efficacy was assessed using the General Self-Efficacy Scale [44]. This scale consists of 10 items rated with a 4-point Likert scale (1 = completely inconsistent; 4 = completely consistent). The higher total score indicated higher self-efficacy. The internal consistency of the scale was acceptable (Cronbach’s α = 0.91).

Perceived social support was assessed using the Perceived Social Support Scale developed by Zimet et al. [45], culturally adapted for Chinese contexts by Jiang [46]. This scale consists of 12 items and uses a 7-point Likert scale (1 = strongly disagree; 7 = strongly agree) to rate. The total score was adopted, with higher scores indicating stronger perceived social support. The Cronbach’s α of this scale in this measurement was 0.93.

Employment confidence was measured by the item referring to the survey of employment confidence conducted by Lu [14]. Participants were asked to evaluate their employment prospects with a 5-point Likert scale (1 = strongly unoptimistic; 5 = strongly optimistic). The higher score indicated higher employment confidence.

### Data analysis

Descriptive statistical analysis for general employment confidence, reliability analysis, and correlation analysis were conducted by SPSS Version 23.0. Meanwhile, the main hypothesized chain mediation model (subjective social class → perceived social support → self-efficacy → employment confidence) was tested by the PROCESS macro in SPSS [47].

## Results

### Common method bias

Harman’s one-factor analysis was used to assess common method bias [48]. All items of variables (subjective social class, perceived social support, self-efficacy, and employment confidence) were subject to exploratory factor analysis to see whether one common factor explained the majority of the covariance. The results revealed that there was a total of four factors with eigenvalue greater than 1, and the maximum explanation of the first factor was 35.94%, which is no more than 40%. Furthermore, by applying the unmeasured latent method construct (ULMC) approach to compare the differences in model fit indices after incorporating the common method factor, it was found that the improvement in relative fit indices was less than 0.1(ΔCFI = 0.069, ΔTLI = 0.066), and the decrease in absolute fit indices was below 0.05(ΔSRMR = 0.028, ΔRMSEA = 0.022), indicating that the model did not exhibit significant improvement [49]. Namely, there was no obvious common method bias in this study.

### Characteristics of college students’ employment confidence

Pearson correlation analysis showed that subjective social class, perceived social support, self-efficacy, and employment confidence were significantly correlated with each other *ps* < 0.01. Subjective social class, perceived social support, and self-efficacy could significantly and positively predict employment confidence. Moreover, employment confidence was significantly correlated with the category and grade of students (*p* < 0.05). The significance of the correlation coefficient between all variables is provided in Table 1.

**Table 1:** Correlation between variables of interest

number	variable	M	SD	1	2	3	4	5	6
1	Gender	1.673	0.469						
2	Grade	1.902	0.774	-0.084*					
3	Student category	1.743	0.437	-0.312**	-0.025				
4	Subjective social class	4.578	1.576	0.213**	0.000	-0.126**			
5	Perceived social support	63.555	11.847	0.105*	-0.002	-0.142**	0.225**		
6	Self-efficacy	27.240	4.969	-0.190**	0.041	0.111**	0.221**	0.309**	
7	Employment confidence	3.407	0.905	0.074	0.092*	-0.263**	0.247**	0.363**	0.476**

Notes: Code for grade: 1 = freshman, 2 = sophomore, and 3 = junior; the code for student category: 1 = public-funded normal students, 2 = other college students. * *p* < 0.05; ** *p* < 0.01

Because there is no significant correlation between gender and employment confidence, it can be seen that gender has no significant effect on the employment confidence of college students. We conducted independent-samples t-test on the scores of public-funded normal students and other college students to analyze the influence of distinct identity (Public-funded normal student) on employment confidence. The results showed that public-funded normal students’ employment confidence (*M* = 3.812, *SD* = 0.807) was significantly higher than that of other college students (*M* = 3.268, *SD* = 0.896) ((*t* (598) = 6.662, *p* < 0.05, *d* = 0.54).

One-way ANOVA was used to explore the differences in employment confidence among different grades. The results showed that grade could significantly affect employment confidence of college students, *F*(2, 597) = 5.367, *p* < 0.01, *η*^2^ = 0.026; The results of post hoc tests indicated that the employment confidence of freshmen (*M* = 3.228, *SD* = 0.061) was significantly lower than that of sophomores (*M* = 3.566, *SD* = 0.058), *p* < 0.01, marginal significantly lower than that of juniors (*M* = 3.412, *SD* = 0.072), *p* = 0.053; there is no significant difference in employment confidence between sophomores and juniors.

### Test of the main hypothesized chain mediation model

Mediation analysis was used to verify the influence mechanism of subjective social class on college students’ employment confidence. We conducted stepwise regression analyses [50] to test a series of mediating effects in which gender, grade, and student category were utilized as control variables, and other variables were standardized before, and the corresponding results are shown in Table 2. The subjective social class could positively predict employment confidence (*β* = 0.226, *p* < 0.001), which supported hypothesis (1) The subjective social class could positively predict perceived social support (*β* = 0.206, *p* < 0.001), and perceived social support could positively predict employment confidence (*β* = 0.168, *p* < 0.001), which supported hypothesis (2) Subjective social class positively predicted self-efficacy (*β* = 0.218, *p* < 0.001), and self-efficacy also positively predicted employment confidence (*β* = 0.447, *p* < 0.001), which supported hypothesis (3) Perceived social support positively predicted self-efficacy (*β* = 0.300, *p* < 0.001), and thus, hypothesis 4 was supported.

**Table 2:** Regression analyses for the testing of mediating effects

Variable	Employment confidence			Perceived social support			Self-efficacy			Employment confidence		
	β	SE	t	β	SE	t	β	SE	t	β	SE	t
Subjective social class	0.226	0.039	5.738^***^	0.206	0.041	5.057^***^	0.218	0.039	5.653^***^	0.067	0.035	1.898
Perceived social support	-	-	-	-	-	-	0.300	0.038	7.894^***^	0.168	0.035	4.733^***^
Self-efficacy	-	-	-	-	-	-	-	-	-	0.447	0.036	12.268^***^
*R* ^*2*^	0.125			0.065			0.198			0.370		
*F*	21.216^***^			10.293^***^			29.406^***^			58.005^***^		

***p < 0.001; ** *p* < 0.01; * *p* < 0.05

**Fig. 2:**
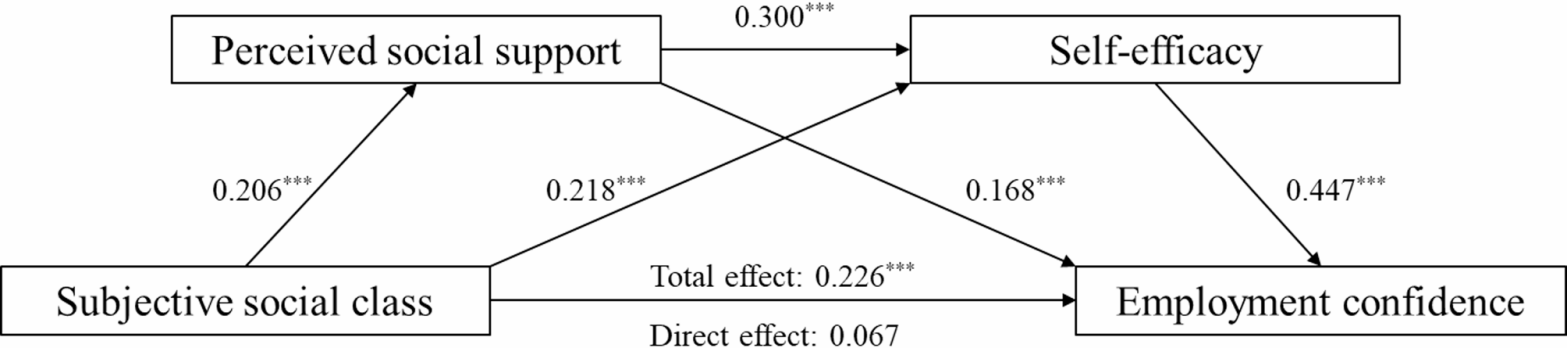
The chain mediation model of the impact of subjective social class on employment confidence

Further, the bootstrap method (based on 5000 bootstrap samples) in the PROCESS macro for SPSS was performed to test the statistical significance of the chain mediating effect. We calculate the 95% confidence interval (CI), which could indicate a significant mediating effect when ‘0’ does not fall within this confidence interval; otherwise, the opposite is true [51]. As shown in Table 3, the results revealed that the direct effect of subjective social class on employment confidence was not significant, with a total effect size of 0.067 (95% CI [-0.002, 0.135]). Perceived social support and self-efficacy had a significant mediating effect between subjective social class and employment confidence, with a total effect size of 0.159 (95% CI [0.111, 0.212]). Specifically, the indirect effect of subjective social class → perceived social support → employment confidence was0.035, with 95% CI [0.014, 0.061], suggesting a significant mediating effect and confirming hypothesis 2; the indirect effect of subjective social class → self-efficacy → employment confidence was 0.097, with 95% CI [0.058, 0.140], suggesting a significant mediating effect and confirming hypothesis 3; the chain mediating effect of subjective social class → perceived social support → self-efficacy → employment confidence was 0.028, with 95% CI [0.014, 0.045], suggesting a significant chain mediating effect and confirming hypothesis 4. Thus, the hypothesized chain mediation model could be confirmed (Fig. 2).

**Table 3:** Results of chain mediating effect

	Effect size	The Boot standard error	95% confidence interval		Ratio of indirect to total effect
			Lower limit	Upper limit	
Total effect	0.226	0.039	0.149	0.303	-
Direct effect	0.067	0.035	-0.002	0.135	-
Total indirect effect	0.159	0.026	0.111	0.212	70.35%
Ind1	0.035	0.012	0.014	0.061	15.49%
Ind2	0.097	0.021	0.058	0.140	42.92%
Ind3	0.028	0.008	0.014	0.045	12.39%

Ind1 is the mediation effect model of Subjective social class, → Perceived social support →Employment confidence; Ind2 is the mediation effect model of Subjective social class → Self–efficacy → Employment confidence; Ind3 is the mediation effect model of Subjective social class → Perceived social support → Self-efficacy → Employment confidence

## Discussion

### College students’ employment confidence varies notably by demographic characteristics

Current research results revealed that there was a significant difference in employment confidence among various categories of college students (public-funded normal students/other college students). Namely, the employment confidence among public-funded normal students is substantially higher than that of other college students. We attributed this result to the preferential policies for public-funded normal students. By China’s policy on public-funded normal university education, public-funded normal students could receive living expense allowances and tuition waivers and have promised to serve in primary and secondary schools for at least six years. The government would allocate premium resources to enhance the teaching quality for public-funded normal students. Local governments should make overall plans to ensure that each public-funded normal student would be implemented in a particular school and position through a two-way selection or guaranteed arrangement [52]. Consequently, the career goal for public-funded normal student was well-defined, and their employment rate approached 100%, accompanied by a high level of employment satisfaction [53]. While other college students endured higher education costs and confronted more intense employment competition and pressure, which consequently resulted in lower employment rates among them [54]. Thus, we contend that the policy initiatives have helped public-funded normal students access more resources and reduce employment pressure, boosting their employment confidence.

The research also identified significant disparities in employment confidence across different grade levels. Specifically, the employment confidence of freshmen was significantly lower than that of sophomores and marginally significantly lower than that of juniors. This is not entirely consistent with previous studies. Previous research has indicated that sophomores exhibit the lowest level of employment confidence, followed by freshmen, seniors, and juniors in ascending order [55]. Another study has demonstrated that undergraduate students’ employment confidence is at its nadir during the freshman year and reaches its zenith in the junior year [56]. Overall, it is a consistent conclusion that freshmen have relatively low confidence in employment. It might be because freshmen have realized severe employment situations since they came to the university. However, their professional ability and comprehensive quality are insufficient, and their career guidance is limited, which inevitably leads to vague career planning and a lack of employment confidence. The present study showed that junior students have lower employment confidence than sophomores, consistent with the ‘diving board theory’ identified by Beaumont [54]. Namely, despite perceiving increased employability as they progress academically, students’ employment confidence decreases, especially in their junior and senior years. It was due to their imminent entry into the job market, which afforded them a more acute awareness of the uncertainties in employment [54, 56]. The ‘diving board theory’ also elucidates why third-year students exhibit lower employment confidence compared to second-year students in the current study.

### The influence mechanism of subjective social class on employment confidence

This study confirms that subjective social class positively influences college students’ employment confidence, and this effect is mediated through the chain mediation of perceived social support and self-efficacy. Since resources and support have direct impacts on problem-solving abilities and personal development [57], subjective social class rooted in material foundations means that groups with higher subjective social class typically have relatively sufficient educational resources and fewer developmental constraints in pursuing self-determined goals. They tend to form stronger competitiveness, enhanced problem-solving capabilities, and greater confidence.

From a cognitive perspective on social class, class-induced differences in cognitive tendencies and self-concept influence individuals’ perceptions and behaviors. Culturally, social class functions as a cultural framework where class-based cultural backgrounds shape individuals’ values and behavioral scripts [11, 58, 59], indicating multidimensional indirect pathways through which social class affects individual perceptions and behaviors. Therefore, individuals’ perceptions of their social support (such as perceived social support) and components of core self-evaluations (like self-efficacy) [45, 60] may mediate the impact of subjective social class on employment confidence. The current study substantiated this hypothesis. College students with higher subjective social class gain more resources from privileged upbringing environments, perceive stronger support during difficulties, develop positive self-evaluations, exhibit higher self-esteem and self-efficacy, and ultimately demonstrate greater employment confidence [11]. Conversely, those with lower subjective social class experience a reduced sense of control, perceive more long-term constraints, hold less optimistic future expectations and self-assessments, and consequently lack confidence [61]. The influence of subjective social class on college students’ employment confidence stems from its long-term effects on individual cognition and self-concept development.

Regarding why perceived social support influences self-efficacy rather than the reverse—particularly given self-efficacy’s stability as an individual trait—we propose two explanations. First, subjective social class inherently originates from long-term socioeconomic foundations and living environments, exhibiting relative stability. The sustained advantages of higher-class families in material resources and educational/emotional investments stably enhance college students’ perceived instrumental and emotional support, making their perceived social support a stable factor that gradually shapes self-efficacy over time. Second, Social Cognitive Theory explicitly confirms that external support influences self-efficacy. As perceived social support encompasses both instrumental (e.g., economic) and emotional (e.g., care) dimensions reflecting support quality [21, 22], previous research consensus supports this directional relationship rather than the reverse [62, 63].

### Cultivation enlightenment of employment confidence

The results of the present study provided some implications for the cultivation of college students’ confidence in employment. Firstly, we should actively pay attention to the employment confidence of low social-class college students. Considering that low social-class college students would face more comprehensive questions, such as the lack of available family resources, weak psychological resistance to stress, and insufficient self-confidence, we need to focus more on the inner selves of low social-class college students rather than simply provide financial help. By strengthening home-school cooperation and collaborative education, we could help students develop more positive self-concepts and improve their confidence in employment. Secondly, we should emphasize the cultivation process to enhance college students’ self-efficacy. There is a prolonged development of employment confidence for college students, which is closely related to their self-efficacy during the university. It is important to strengthen the cultivation process, involving teaching, student activities, and practical exercises during school. Simultaneously, teachers should profusely admire students’ positive behavior and progress and guide students to consolidate their self-efficacy, thereby shaping more positive mental qualities. The third is to pay attention to students’ sense of social support in the process of employment. Students would encounter lots of problems and pressures in the process of employment. If the means of employment assistance are more universal instead of targeted, it will reduce students’ sense of support. Therefore, considering individual differences among college students, the employment support provided by schools is not limited to employability enhancement training or economic subsidies, but can also provide targeted support, to a certain extent, according to the actual needs of students by demand investigation, so as to effectively improve students’ sense of social support.

### Limitations and future research directions

The present study offers valuable insights into understanding the employment confidence of college students and exploring methods to enhance it, yet there still remain some limitations. Firstly, Subjective and objective social class are not entirely congruent. The present study has not taken into account the influence of objective social class. Future research should separately analyze and compare the differential impacts of subjective and objective social class to enhance the validity and robustness of the research. Secondly, the study had a relatively small sample size, with participants primarily recruited from two universities. The size and proportion of different demographic groups were not adequately balanced, which may limit the generalizability of the findings and hinder further analysis of specific differences in mediation models. Thirdly, to minimize the impact of job-hunting experience on employment confidence, this study primarily focuses on freshmen, sophomores, and juniors, excluding seniors. Fourth, in terms of research methodology, this study lacks qualitative data to further analyze the factors influencing employment confidence. Future research should adopt a mixed-methods approach combining qualitative and quantitative analyses to conduct more in-depth and comprehensive explorations into how college students’ job-seeking experiences and their perception of career barriers impact employment confidence. It is also worthwhile to analyze how personal characteristics, environmental factors, and individual behaviors influence such confidence by increasing the sample size and balancing demographic groups in order to provide deeper insights.

## Conclusion

The present study found that the overall employment confidence of college students was positive. The employment confidence of public-funded normal students was significantly higher than that of other college students; both sophomore and junior students’ employment confidence surpassed freshmen’s. Meanwhile, the four hypotheses were confirmed. Namely, the subjective social class of college students could directly and positively predict their employment confidence and also could affect employment confidence through the chain mediating effect of individual perceived social support and self-efficacy.

## Electronic supplementary material

Below is the link to the electronic supplementary material.

Supplementary Material 1

Supplementary Material 2

Supplementary Material 3

## Data Availability

All data generated or analyzed during this study and materials used in this study are included in supplementary information files.

## Abbreviations

ULMC: Unmeasured Latent Method Construct
