# Supplementary figures and images for: The influence of subjective social class on employment confidence: the chain mediating effect of perceived social support and self-efficacy
Source: BMC Psychol. 2025 Sep 10;13:737. doi: 10.1186/s40359-025-02861-3 (PMC12421765; doi:10.1186/s40359-025-02861-3)

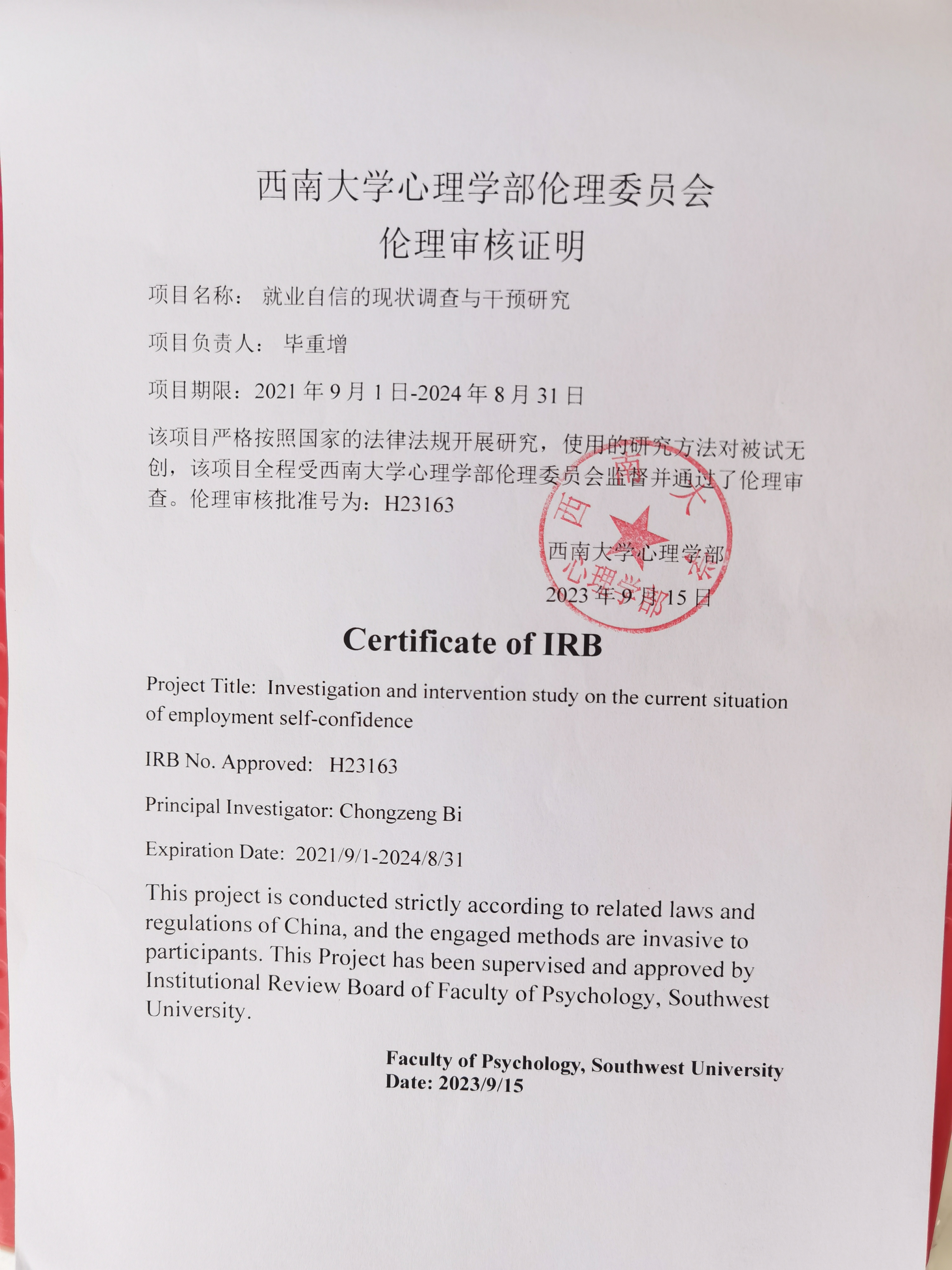

Supplement: Supplementary file 1 — Supplementary Material 1 [file 40359_2025_2861_MOESM1_ESM.jpg]
