# Supplementary material for: The influence of subjective social class on employment confidence: the chain mediating effect of perceived social support and self-efficacy
Source: BMC Psychol. 2025 Sep 10;13:737. doi: 10.1186/s40359-025-02861-3 (PMC12421765; doi:10.1186/s40359-025-02861-3)
Supplement: Supplementary file 3 — Supplementary Material 3 [file 40359_2025_2861_MOESM3_ESM.docx]

Welcome to participate in the questionnaire about college students' status at school and employment perception. Please feel free to fill it out. This questionnaire adopts anonymous survey, and all the information you fill in will be used as research and will not be leaked.

Please note that there is no right or wrong answer to any of the questions. You can simply respond based on your true and primary reaction, without deep consideration. Thank you for your participation!

Gender: (male female) Age:

Grade: (freshman sophomore junior senior)

publicly-funded normal student or not: (Yes No)

Ⅰ. Subjective social class measurement (MacArthur scale):

| Imagine that the ladder on the right represents the different social classes in society. Rate where your family is currently in the social class.And put "√" on the corresponding number：  The "10" at the top of the scale corresponds to people in the highest social class, who are in the best living conditions, with the highest incomes, the highest educational attainment and the most decent jobs；  The"01" at the bottom of the scale corresponds to people in the lowest social class, who are in the worst living conditions, with the lowest incomes, the lowest educational attainment and the least decent jobs. | 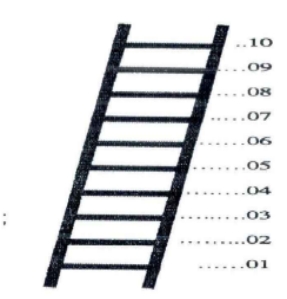 |
| --- | --- |

Ⅱ. Perceived Social Support Scale：

|  | Strongly disagree | disagree | a little disagree | uncertain | a little agree | agree | Strongly agree |
| --- | --- | --- | --- | --- | --- | --- | --- |
| 1. There are people (teachers, relatives, classmates) who are there for me when I have a problem. |  |  |  |  |  |  |  |
| 2. There are people (teachers, relatives, classmates) with whom I have been able to share happiness and sorrow. |  |  |  |  |  |  |  |
| 3. My family can help me in a concrete way. |  |  |  |  |  |  |  |
| 4. I can get emotional help and support from my family when I need it. |  |  |  |  |  |  |  |
| 5. Some people (teachers, relatives, classmates) are a real source of comfort when I am in trouble. |  |  |  |  |  |  |  |
| 6. My friends can really help me. |  |  |  |  |  |  |  |
| 7. I can count on my friends in times of trouble. |  |  |  |  |  |  |  |
| 8. I can talk to my family about my problems. |  |  |  |  |  |  |  |
| 9. My friends can share happiness and sorrow with me. |  |  |  |  |  |  |  |
| 10. There are certain people in my life (teachers, relatives, classmates) who care about my feelings. |  |  |  |  |  |  |  |
| 11. My family is willing to help me make decisions. |  |  |  |  |  |  |  |
| 12. I can discuss my problems with my friends. |  |  |  |  |  |  |  |

Ⅲ. General Self-Efficacy Scale:

|  | completely inconsistent | a little  inconsistent | a little consistent | completely consistent |
| --- | --- | --- | --- | --- |
| 1. I can always solve problems if I try my best. |  |  |  |  |
| 2. Even if others oppose me, I can still get what I want. |  |  |  |  |
| 3. It's easy for me to stick to my ideals and achieve my goals. |  |  |  |  |
| 4. I am confident that I can deal with any unexpected event effectively. |  |  |  |  |
| 1. With my talents, I'm sure I can cope with unexpected situations. |  |  |  |  |
| 6. If I put in the necessary effort, I'm sure I can solve most of the problems. |  |  |  |  |
| 7. I can face difficulties calmly because I trust my ability to deal with problems. |  |  |  |  |
| 8. When faced with a difficult problem, I can usually find several solutions. |  |  |  |  |
| 9. When in trouble, I can usually think of some way to deal with it. |  |  |  |  |
| 10. Whatever comes my way, I can handle it. |  |  |  |  |

Ⅳ. Employment confidence:

|  | Very unoptimistic | a little unoptimistic | uncertain | a little optimistic | Very optimistic |
| --- | --- | --- | --- | --- | --- |
| How you feel about your job prospects |  |  |  |  |  |
